# Identification and distribution of a GABA receptor mutation conferring dieldrin resistance in the malaria vector *Anopheles funestus* in Africa

**DOI:** 10.1016/j.ibmb.2011.03.012

**Published:** 2011-07

**Authors:** Charles S. Wondji, Roch K. Dabire, Zainab Tukur, Helen Irving, Rousseau Djouaka, John C. Morgan

**Affiliations:** a Vector Group, Liverpool School of Tropical Medicine, Pembroke Place, Liverpool L3 5QA, United Kingdom; b Burkina Faso Institut de Recherche en Science de la Santé, Direction Régionale, de Bobo-Dioulasso, Burkina Faso; c Centre Muraz, BP 390, Bobo-Dioulasso, Burkina Faso; d Bayero University, Kano, Nigeria; e International Institute of Tropical Agriculture, 08 BP 0932 Cotonou, Benin

**Keywords:** Insecticide resistance, *Anopheles funestus*, Dieldrin resistance, GABA receptor, Malaria

## Abstract

Growing problems of pyrethroid resistance in *Anopheles funestus* have intensified efforts to identify alternative insecticides. Many agrochemicals target the GABA receptors, but cross-resistance from dieldrin resistance may preclude their introduction.

Dieldrin resistance was detected in *An. funestus* populations from West (Burkina Faso) and central (Cameroon) Africa, but populations from East (Uganda) and Southern Africa (Mozambique and Malawi) were fully susceptible to this insecticide. Partial sequencing of the dieldrin target site, the γ-aminobutyric acid (GABA) receptor, identified two amino acid substitutions, A296S and V327I. The A296S mutation has been associated with dieldrin resistance in other species. The V327I mutations was detected in the resistant sample from Burkina Faso and Cameroon and consistently associated with the A296S substitution. The full-length of the *An. funestus* GABA-receptor gene, amplified by RT-PCR, generated a sequence of 1674 bp encoding 557 amino acid of the protein in *An. funestus* with 98% similarity to that of *Anopheles gambiae*. Two diagnostic assays were developed to genotype the A296S mutation (pyrosequencing and PCR-RFLP), and use of these assays revealed high frequency of the resistant allele in Burkina Faso (60%) and Cameroon (82%), moderate level in Benin (16%) while low frequency or absence of the mutation was observed respectively in Uganda (7.5%) or 0% in Malawi and Mozambique.

The distribution of the *Rdl^R^* mutation in *An. funestus* populations in Africa suggests extensive barriers to gene flow between populations from different regions.

## Graphical abstract

**Figure:**
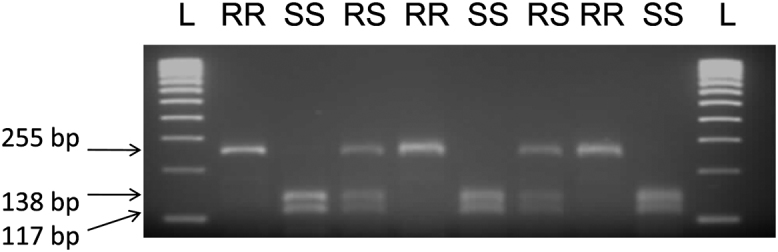

**Highlights:**

► Dieldrin resistance detected in *An. funestus* from West and Central Africa. ► Identification of the A296S conferring dieldrin resistance after sequencing. ► V327I mutation detected in resistant samples and associated with the A296S mutation. ► Two diagnostic assays were developed to genotype the A296S mutation. ► High frequency of *Rdl^R^* in West Africa but complete absence in southern Africa.

## 1 Background

The mosquito *Anopheles funestus* is a major vector of malaria throughout much of sub-Saharan Africa. Its efficiency as a vector is partially conferred by its highly anthropophilic and endophilic behaviours and in many places, parasite infection rates of *An. funestus* even exceed those of *Anopheles gambiae* (Coetzee and Fontenille, 2004).

Control of *An. funestus* as well as other malaria vectors relies extensively on the use of insecticides, either as insecticide treated materials or as indoor residual sprays (IRS). Unfortunately, *An. funestus* is increasingly developing resistance across Africa to different classes of insecticides used in public health, such as pyrethroids, carbamates and DDT (Brooke et al., 2001; Casimiro et al., 2006; Cuamba et al., 2010; Morgan et al., 2010). There are alternative agrochemicals, such as fipronil that could be introduced but the potential for cross-resistance from existing mechanisms segregating in field populations needs to be established. One of the first cases of insecticide resistance reported in this species was from Burkina Faso, where resistance was found to dieldrin, a cyclodiene abundantly used in Africa in the 1960s (Hamon et al., 1968). Dieldrin resistance was also reported in *An*. *funestus* from Cameroon, Benin, Nigeria and Mali (Service, 1960; Toure, 1982; Brown, 1986). Recent studies have shown that dieldrin resistance remains high in *An. funestus* populations from Burkina Faso despite the fact that cyclodienes are no longer used in public health control programs (Dabire et al., 2007), but the distribution of this resistance throughout the rest of the continent is unknown. Understanding the factors explaining the persistence of high levels of resistance against cyclodienes in *An. funestus* as well as the geographical distribution of this resistance across the continent could provide useful information for management. Characterising the mechanism conferring this resistance and assessing its distribution across Africa will be a first step towards this goal.

Resistance to dieldrin in many insects, including *An. gambiae* and the fruit fly *Drosophila melanogaster*, is conferred by a mutation in the gene coding for a subunit of the γ-aminobutyric acid (GABA) receptor, a chloride channel (Ffrench-Constant et al., 1993; Thompson et al., 1993; Du et al., 2005). This GABA receptor is made up of five subunits. Each subunit has an extracellular cysteine loop and four transmembrane domains (M1–M4). The M2 transmembrane domain contains a conserved alanine residue (position 302 in *D*. *melanogaster*, 296 in *An. gambiae* (Du et al., 2005)). Two amino acid substitutions (A302S and A302G) at this residue have been associated with dieldrin resistance in various insect species. In mosquitoes, the A296G substitution has been observed in *An. gambiae* (Du et al., 2005) while the A296S substitution was associated with dieldrin resistance in *Anopheles arabiensis*, *Anopheles stephensi* and *Aedes aegypti* (Ffrench-Constant et al., 2000; Du et al., 2005). The underlying mechanism conferring dieldrin resistance in *An. funestus* is unknown.

Here we report the detection of the *Rdl* mutation for the first time in *An. funestus* and present a geographical distribution of this mutation in *An. funestus* populations from different regions of Africa after designing two diagnostic assays to genotype this mutation.

## 2 Methods

### 2.1 Mosquito collection

Blood fed, gravid or half gravid *An. funestus* adult females were collected inside houses using aspirators and torches in six countries in Africa between 06 and 12 AM. The collections were performed in the following sites: Tororo (0°45′N, 34°5′E) in Uganda in November 2009, Soumoussou (11°00′N, 4°02′W) in Burkina Faso in March 2009, Lagdo (9°05′N, 13°40′E) in Cameroon in November 2006, Chikwawa (12°19′S, 34°01′E) in Malawi in April 2010, Chokwe (24°33′S, 33°01′E) in Mozambique in February 2009 and Pahou (6°23′N, 2°13′E) in Benin in March 2010. The females were left to oviposit and F_1_ adults were reared using the protocol recently described (Morgan et al., 2010).

### 2.2 PCR-species identification

All females used for oviposition were morphologically identified as belonging to the *An. funestus* group (Gillies and De Meillon, 1968) and identified to species by PCR (Koekemoer et al., 2002).

### 2.3 Dieldrin susceptibility assay

Susceptibility status to dieldrin was assessed with WHO bioassays using 2–5 day-old F_1_ adults from mass-reared mosquitoes following the WHO protocol (WHO, 1998). Around 20–25 mosquitoes per tube were exposed to 4% dieldrin-impregnated filter papers for 1 h and then transferred to a clean holding tube supplied with a 10% sugar solution. Mortality was determined after 24 h.

### 2.4 *Rdl* amplification and sequencing and analysis

A fragment of exon 7 of the GABA-receptor gene spanning the *Rdl* mutation was initially amplified in 10 resistant mosquitoes from Burkina Faso and five susceptible from Uganda. DNA was extracted using the LIVAK method (Collins et al., 1987) and amplified using the primers previously used for *An. gambiae* and *An. arabiensis*; RDLF, 5′-AGT TTG TAC GTT CGA TGG GTT A-3′ and RDLR, 5′-CCA GCA GAC TGG CAA ATA CC-3′ (Du et al., 2005). The PCR was carried out using 10 pmol of each primer and 10 ng of genomic DNA as template in 25 μl reactions containing 1X Kapa Taq buffer, 0.2 mM dNTPs, 1.5 mM MgCl_2_, 1U Kapa Taq (Kapa biosystems). The cycle parameters were: 1 cycle at 95 °C for 5 min; 35 cycles of 94 °C for 30 s, 57 °C for 30 s and elongation at 72 °C for 30 min; followed by 1 cycle at 72 °C for 10 min. PCR products were purified using Exo-SAP clean up protocol and directly sequenced on both strands using the two primers above. Sequences were aligned using ClustalW (Thompson et al., 1994).

The SuperScript^®^ III One-Step RT-PCR System with Platinum^®^ *Taq* DNA Polymerase from Invitrogen was used to amplify the full-length of the GABA-receptor gene in *An. funestus*. RNA was extracted using the Picopure RNA isolation kit (Arcturus) from a batch of ten dieldrin resistant females from the resistant *An. funestus* population of Soumoussou from Burkina Faso. The following primers, RdlfullF 5′-ATG TCG CTA ACT ATC GAA GTT CCG C-3′ and RdlfullR 5′-TTA CTT CTC CTC GCC CAG CAG CA-3′, based on the *An. gambiae* sequence, were used for the amplification. The RT-PCR was carried out using 10 pmol of each primers and 25 ng RNA as template in 15 μl reactions containing 2× reaction buffer, 1U Superscript enzyme mix (SSIII Platinum Tag Mix). The cycle parameters were: 1 cycle at 55 °C for 30 min and 94 °C for 2 min followed by 30 cycles of 94 °C for 15 s, 58 °C for 30 s and elongation at 68 °C for 1 min; followed by 1 cycle at 68 °C for 5 min. The RT-PCR product was gel-purified using the Qiagen gel purification kit, cloned into the CloneJet vector (Fermentas) and sequenced in both directions.

### 2.5 Pyrosequencing *Rdl* diagnostic assay

A diagnostic assay was designed to detect the *Rdl* resistant allele (*Rdl^R^*) in *An. funestus* populations using the pyrosequencing method. The pyrosequencing method monitors DNA synthesis in real time by recording bioluminescence resulting from a cascade of reactions triggered by the incorporation of a nucleotide. Three sequence-specific primers were designed to genotype the mutation (Table 1) using the software provided by Pyrosequencing AB (http://www.pyrosequencing.com). The sequence to analyze was 5′-T G/T G/C ATTAGGTGT-3′and the dispensation order was 5′-TGCgATAGT-3′. The lower case of nucleotide “g” means it is the negative control and should not be incorporated in the target DNA. A target DNA fragment was first amplified by PCR using the forward and the biotinylated reverse primers. Pyrosequencing reactions were performed as described previously (Wondji et al., 2007, 2008) according to the manufacturer’s instructions using the PSQ 96 SNP reagent Kit (QIAGEN) and the genotype was determined by the pyrosequencer and illustrated on pyrograms.

An additional pyrosequencing diagnostic assay was designed to genotype a mutation inducing an amino acid change in exon 7. The sequencing primer and the sequence to analyse of that mutation are presented in Table 1.

### 2.6 PCR-RFLP for alternative genotyping method

A PCR-RFLP assay was developed as an alternative method to pyrosequencing to genotype the *Rdl* mutation in *An. funestus*. The same primers RDLF and RDLR used above were used to amplify the fragment of 255 bp of Exon 7 using the same PCR conditions. The PCR product was directly digested with HpyCH4V restriction endonuclease (New England Biolabs) during 1 h of incubation at 37 °C. This restriction enzyme cleaves the susceptible allele at the recognition sequence TG^CA producing two fragments detectable on a 2% agarose gel.

### 2.7 Analysis of distribution of *Rdl An. funestus*

The geographical distribution of the *Rdl* mutation was assessed using field collected females (F_0_) from six populations of *An. funestus* in Africa. The geographic structure of the GABA-receptor gene was also assessed using the Genepop 4.0.10 program (Raymond and Rousset, 1995) with the following parameters assessed for each population or between populations: the frequency of *Rdl^R^* allele, heterozygosity, Hardy–Weinberg equilibrium analysis, genotypic differentiation between pair of samples.

## 3 Results

### 3.1 Resistance to dieldrin

WHO bioassays, using mixed F_1_ progeny from field caught females, indicated resistance to dieldrin in *An. funestus* populations from Burkina Faso (*n* = 107) and Cameroon (*n* = 90) with mortality levels of 30 and 20% respectively. Full susceptibility to dieldrin was recorded for populations from Mozambique, Malawi and Uganda. Bioassays were not performed on the population from Benin. This pattern of susceptibility suggests that dieldrin resistance is mainly present in *An. funestus* populations from West to Central Africa.

### 3.2 Detection of mutations associated with dieldrin resistance

A 255 bp fragment was successfully amplified in *An. funestus*. Direct sequencing of the PCR product confirmed that this was exon 7, encoding the M2 transmembrane domain region, of the *An. funestus* GABA-receptor gene (Genbank Accession number: HQ645084).

**Fig. 1:**
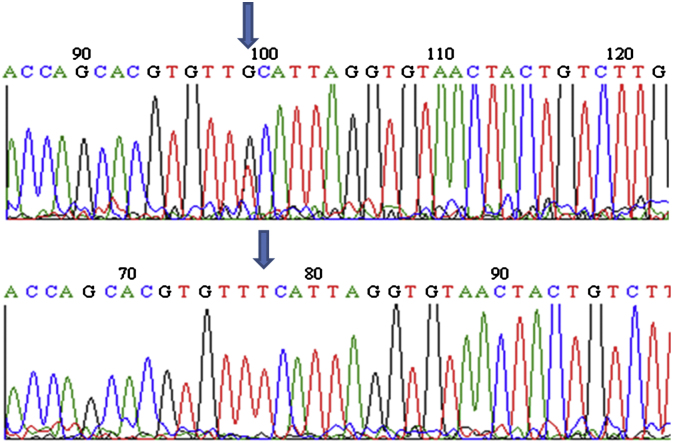
Sequence chromatograms of the fragment of exon 7 of the GABA-receptor gene for a heterozygous and homozygous dieldrin resistant *An. funestus*. The polymorphic site is indicated by an arrow.

A point mutation (GCT to TCT) inducing an amino acid change of alanine to serine (A296S) which confers dieldrin resistance in *An. arabiensis* (Du et al., 2005), was observed in all the dieldrin resistant samples from Burkina Faso, while all of the five susceptible samples sequenced from Uganda contained the GCT codon at position 296 (Fig. 1). The GCT to GGT mutation found in *An. gambiae* and causing the A296G change was not observed in *An. funestus* samples.

Sequence comparison led to the identification of an additional mutation at codon 327 inducing an amino acid change of valine (GTA) to isoleucine (ATA) in the resistant mosquitoes from Burkina Faso. Following the detection of this additional mutation, we attempted to amplify and sequence the full-length of the GABA-receptor gene in this species in order to check whether other non-synonymous mutations could be identified. This resulted in the amplification, by RT-PCR, of a full sequence of the GABA-receptor gene. This 1674 bp fragment (Genbank Accession number: JF460792) is predicted to encode 557 amino acids of the protein contrary to 555 in *An. gambiae*. It corresponds to the isoform B of the three transcripts of the GABA-receptor gene in *An. gambiae* (AGAP006028-RB) and *D. melanogaster* (FBgn0004244). The *An. funestus* cDNA is 96% similar to isoform B of *An. gambiae* while only 93 and 92% similar to isoforms A and C respectively. The *An. funestus* protein sequence is 98% similar to the isoform B protein sequence of *An. gambiae* (AGA006028-PB) with variation of 7 amino acids while only 96% similar to both isoforms A and C.

**Fig. 2:**
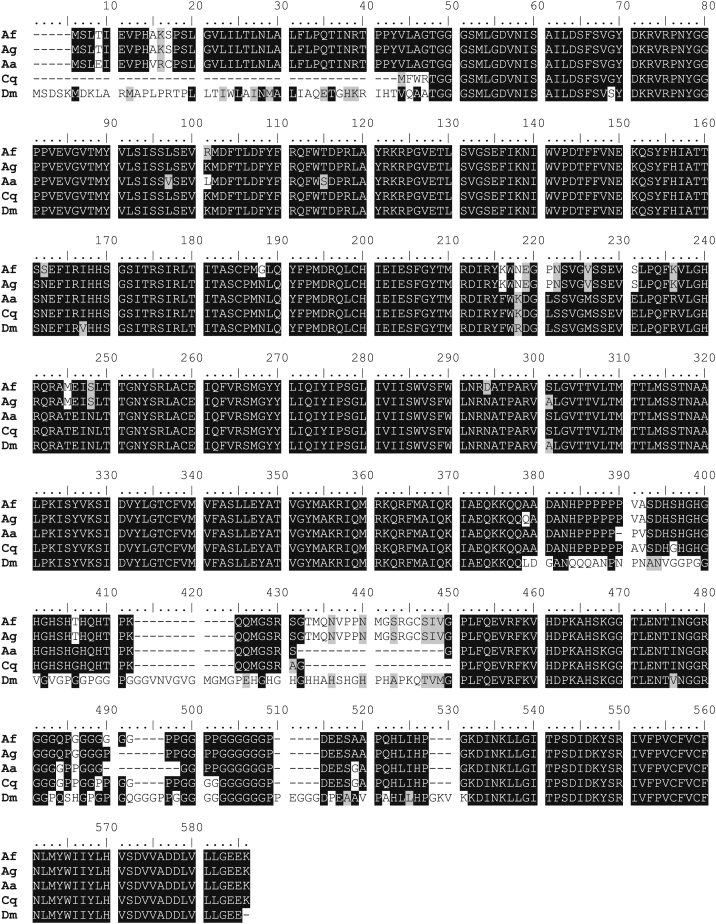
Alignment of amino acid sequences of the GABA-receptor gene between *An. funestus* (Af), *An. gambiae* (Ag), *Ae. aegypti* (Aa), *Cx. quinquefasciatus* (Cq) and *D. melanogaster* (Dm). The A296S *Rdl* mutation is indicated by an arrow while the V327I mutation is indicated and inverted triangle.

A comparison of the amino acid sequences of the GABA-receptor gene between the *An. funestus* sequence and other insect species reveals that the *An. funestus* sequence is closer to that of *An. gambiae* and *Ae aegypti* with 98 and 94% of similarity respectively, than to *Culex quinquefasciatus* and *D. melanogaster* with 84 and 86% similarity respectively (Fig. 2).

### 3.3 Diagnostic assays to genotype the *Rdl* mutation in *An. funestus*

**Fig. 3:**
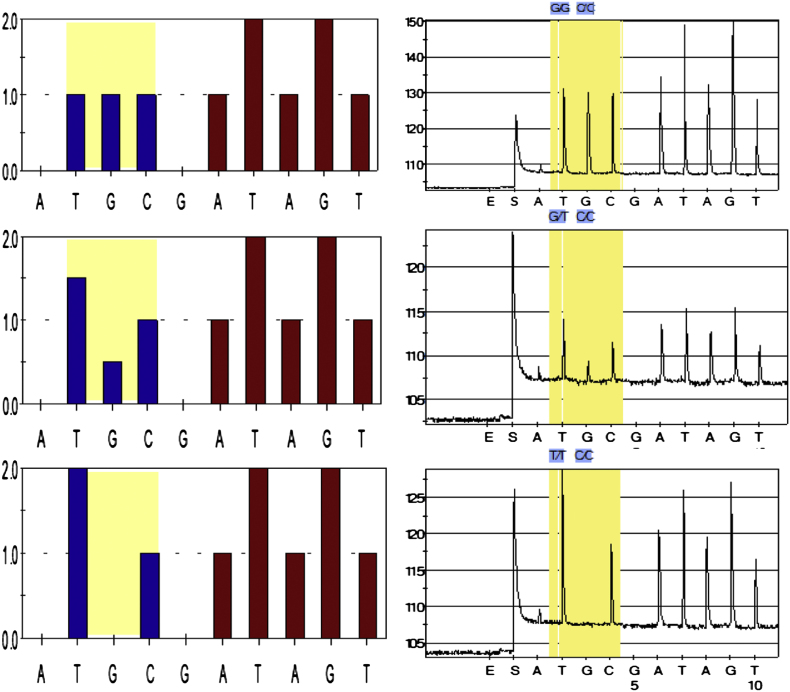
Expected histograms and observed pyrograms of the Rdl mutation pyrosequencing assay.

Two diagnostic assays were designed to genotype the A296S mutation. The pyrosequencing method successfully identified the three genotypes according to the predicted histograms (Fig. 3). In this study, the assay was used to detect the two potential nucleotides (G or T) at the first coding position of the 296 codon in a single reaction. The dispensation order 5′-TGCgATAGT-3′ generated by the pyrosequencer program gives the order in which the nucleotides are added in the reaction, and the height of the peak corresponds to the number of nucleotides. With this principle, the G/G genotype is detected when the peaks of the three first nucleotides T, G and C are of equal height (Fig. 3), while G/T genotype is detected when the height of nucleotide T is 3 times that of G and 1.5 times that of C. The T/T genotype is detected when there is no peak for G nucleotide and the height of the T nucleotide is twice that of nucleotide C. All these three genotypes were reliably scored using this method, as presented in Fig. 3. To further confirm the accuracy of this genotyping method, the 10 mosquitoes directly sequenced for the 255 bp fragment of exon 7 of the GABA receptor were also genotyped by pyrosequencing and there was a perfect agreement between the results.

**Fig. 4:**
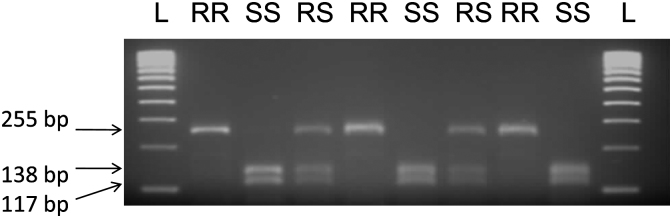
Agarose gel of a PCR-RFLP to detect dieldrin resistance in *An. funestus*. The top band is a 255 bp fragment for resistant mosquitoes while the bottom bands represent the 117 and 138 bp fragments resulting from the restriction digestion by HpyCH4V.

In order to provide a diagnostic assay that could be applied in laboratories without access to a pyrosequencer, we also designed a PCR-RFLP assay to genotype the A296S mutation. This assay accurately detected the three genotypes as confirmed by direct sequencing. After amplification of the 255 bp fragment by PCR, the HpyCH4V restriction endonuclease cuts the susceptible allele to generate two fragments of 117 and 138 bp that co-migrate at the same position on a 2% agarose gel to give one band for G/G genotype (Fig. 4). The resistant allele is not cut by the enzyme and therefore a band of 255 bp is observed on the gel. For G/T heterozygotes two bands are clearly visible, one representing the 255 bp and the other corresponding to the co-migrating 117 and 138 bp fragments.

### 3.4 Geographical distribution of the A296S *Rdl* mutation in Africa

**Fig. 5:**
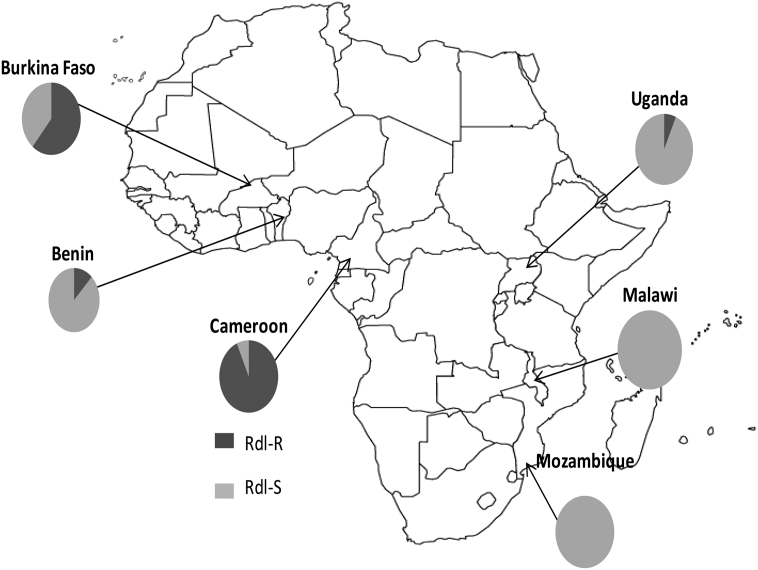
Geographical distribution of the *Rdl^R^* allele in six countries in Africa.

Using the pyrosequencing method we assessed the distribution of the *Rdl^R^* allele in different regions of Africa by genotyping *An. funestus* samples from 6 countries: Burkina Faso, Cameroon, Benin, Uganda, Malawi and Mozambique. The *Rdl^R^* was detected in four out of the six countries with frequencies ranging from 0 to 82% (Fig. 5). Highest frequencies were observed in West (Burkina Faso) and Central Africa (Cameroon) with 60 and 82% respectively but a moderate frequency of 16% was observed in Benin. The *Rdl^R^* allele was also detected in East Africa (Uganda) although at a very low frequency of 7.5% and only in heterozygotes. The *Rdl^R^* allele was not observed in the two southern African countries of Mozambique and Malawi indicating that this allele has probably not yet spread to *An. funestus* population of this region. We observed a significant departure from Hardy–Weinberg equilibrium with heterozygote deficit in the two populations with the highest *Rdl^R^* frequencies from Cameroon (*P* = 0.0115) and Burkina Faso (*P* = 0.0026) (Table 2). Although an excess of heterozygotes was seen in Benin and Uganda, these were not statistically significant.

Because the pyrosequencing method sequences a short fragment of the gene, it offers the opportunity to analyse whether other mutations are present around the target mutation. Taking advantage of this, we also monitored whether there was a C-to-G mutation in codon 296 inducing the alanine to glycine amino acid change as seen in *An. gambiae*. No such mutation was observed at this position for all the samples screened, indicating that only the A296S is present in *An. funestus*.

### 3.5 Geographic structure of *Rdl* resistance gene in *An. funestus* in Africa

Samples from Burkina Faso and Cameroon with a high frequency of *Rdl^R^* allele showed no genotypic differentiation (*P* > 0.5) between them. But when compared to the other 4 samples, highly significant differentiations were observed, particularly against samples from southern Africa such as Mozambique and Malawi. There was no genotypic differentiation between the samples from Southern Africa (Mozambique and Malawi) as expected. The observed pattern of geographic structure in the *Rdl* gene is only disrupted by the lower *Rdl^R^* frequency observed in Benin compared to Burkina Faso and Cameroon. However, the dieldrin resistance seems to be localized in West Africa, at a low frequency in East Africa and absent in southern Africa. As resistance to dieldrin has been detected in *An. funestus* since the 1960s, this may be an indication of the existence of barriers to gene flow between populations of *An. funestus* between these regions of Africa.

### 3.6 Analysis of the correlation between the V327I and the A296S *Rdl* mutations

**Fig. 6:**
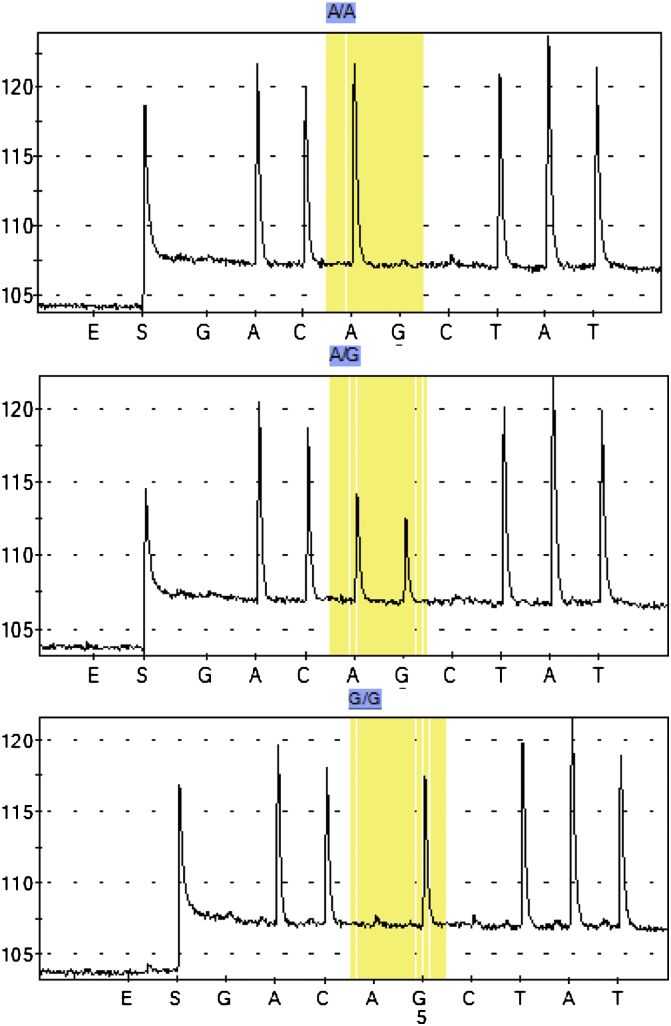
Observed pyrograms of the V327I mutation for the pyrosequencing assay.

**Fig. 7:**
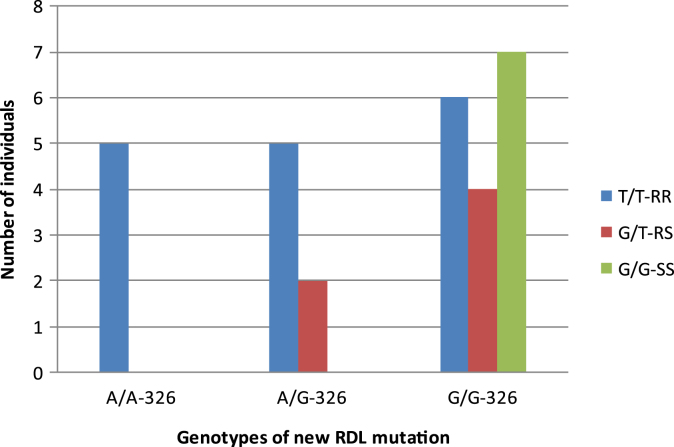
Association of the V327I genotypes and those of the *Rdl* mutation A296S.

The V327I mutation, genotyped in the same samples as for the A296S mutation (Fig. 6), was only detected in Cameroon and Burkina Faso populations, where the A296S is present at high frequency. The frequency of the V327I mutation is lower than that of the A296S (Table 2) with 17.25 and 21% in Cameroon and Burkina Faso respectively. The homozygote A/A genotype inducing the valine to isoleucine change at codon 327 is only found in homozygote T/T individuals with the alanine to serine replacement at codon 296 (Fig. 7). The heterozygote A/G genotype at codon 327 is associated with the homozygote resistant T/T and heterozygote G/T at codon 296 but was not found in susceptible homozygote G/G individuals (Fig. 7). The homozygote G/G genotype for the ‘wild type’ valine codon at 327 was observed with all the three genotypes of the 296 mutation.

## 4 Discussion

The dieldrin resistance observed in the *An. funestus* population from Soumousso in Burkina Faso confirms previous reports of resistance in this country (Hamon et al., 1968; Dabire et al., 2007). Dieldrin resistance was also reported in *An. funestus* from Cameroon, Benin, Nigeria and Mali (Service, 1960; Toure, 1982; Brown, 1986). Bioassays in West Africa (Burkina Faso) and Central Africa (Cameroon), revealed resistance, but there was no dieldrin resistance in East Africa (Uganda) and southern Africa (Malawi and Mozambique) with 100% mortality recorded for each of these countries. This indicates that dieldrin resistance in *An. funestus* is geographically restricted.

The high level of dieldrin resistance in field populations of *An. funestus* in West Africa which is also seen in *An. gambiae* and *An. arabiensis* (Etang et al., 2003; Du et al., 2005) despite the fact that cyclodienes are no longer used for control programs is significant. Rowland (1991a; 1991b) demonstrated that the dieldrin resistance gene confers a significant fitness cost in two *Anopheles* species, *An. gambiae* and *An. stephensi*, impacting their behaviour, activity and mating competitiveness. Therefore, in the absence of dieldrin selection, a reversion of the resistance would be expected. This was observed in Northern Nigeria where, 6 years after the discontinuation of dieldrin spraying, the *An. gambiae* population reverted to susceptibility (Hamon and Garrett-Jones, 1963). The same reversion has been documented for *An. culicifacies* in India (Bhatia, 1963). Therefore, the persistence of dieldrin resistance in *An. funestus* in West Africa may be the result of the use of agrochemicals targeting the GABA receptor in the agricultural sector, rather than reflecting a lack of fitness cost. Agricultural use of fipronil or lindane was suggested recently to explain the high *Rdl^R^* frequency in *Culex pipiens* and *Aedes albopictus* populations in La Reunion (Tantely et al., 2010). This could also be the case in Burkina faso as these insecticides are used by cotton farmers (R. Dabire, Unpublished data). Understanding the factors explaining the persistence of high level of resistance against cyclodienes in *An. funestus* could provide useful information for resistance management. In *An. gambiae*, the dieldrin resistance gene is also associated with the 2La chromosomal inversion (Brooke et al., 2000). This is a highly stable inversion polymorphism that limits crossing-over and would contribute to preserve the dieldrin mutation in a population even without any selection pressure and despite a fitness cost. The 2L chromosome in *An. gambiae* corresponds to the 3R in *An. funestus* which contains 3 inversions (3Ra, 3Rb and 3Rd)(Sharakhov et al., 2004). Although the GABA-receptor gene has not been physically mapped in *An. funestus*, it is not excluded that it could also be located in one of these 3 inversions reducing crossing-over and leading to the preservation of this mutation in the population as seen in *An. gambiae* (Brooke et al., 2000, 2002).

The *Rdl* mutation observed in *An. funestus* is the A296S mutation previously reported in *An. arabiensis*, rather than the A296G seen in *An. gambiae* (Du et al., 2005). DNA sequence conservation around this mutation means that diagnostic tools developed for *An. funestus* could also be used to genotype *Rdl* in *An. arabiensis* (PCR-RFLP or pyrosequencing) and *An. gambiae* (pyrosequencing only).

Only one GABA-receptor isoform (similar to isoform B in *An. gambiae*) was found in *An. funestus*, compared to three in *An. gambiae*, but more sequencing will need to be carried out to assess whether the two other isoforms of the GABA receptor (B and C) are also present in *An. funestus*. Analysis of the genomes of *Cx. quinquefasciatus* and *Ae. aegypti* has revealed only one GABA-receptor gene isoform for these species, while three transcripts are observed in *D. melanogaster*.

The distribution of the *Rdl^R^* mutation in Africa correlated perfectly with the resistance level to dieldrin with a high frequency of this mutation in West (Burkina Faso) and Central Africa (Cameroon), while a low frequency was observed in East Africa (Uganda) and complete absence in the two southern African countries (Malawi and Mozambique). This discontinuous pattern of dieldrin resistance in *An. funestus* populations in Africa is similar to the resistance pattern observed for pyrethroid, carbamate and DDT in this species. Indeed while there is only pyrethroid and carbamate resistance in southern Africa populations and full susceptibility to DDT (Brooke et al., 2001; Casimiro et al., 2006; Cuamba et al., 2010), there is both pyrethroid and DDT resistances in East Africa (Uganda) (Morgan et al., 2010), and resistance to all three insecticide classes has been observed in West Africa (Okoye et al., 2008). This suggests a significant restriction of gene flow between *An. funestus* populations across Africa, with a probable existence of subdivision as suggested by Michel et al. (2005), who on the basis of microsatellite markers, indicated that *An. funestus* populations in Africa could be split in 3 main subgroups (Western, Central and southern groups). Similar observations were also made by Garros et al. (2004). The pattern of resistance and the distribution of the *Rdl^R^* allele support this subdivision in *An. funestus* populations in Africa, and the *Rdl^R^* mutation could be used as an indicator of gene flow in *An. funestus* populations.

The analysis of the geographical structure of the *Rdl* gene in *An. funestus* indicated a heterozygotes deficit in resistant populations of Cameroon and Burkina Faso. This could be due to the possible existence of an assortative mating between RR and SS as observed by (Rowland, 1991a) in *An. gambiae* and *An. stephensi* where he found that RR males tend to mate preferentially with RR females.

The new valine to isoleucine amino acid substitution detected at codon 327 in the M2 transmembrane domain as the A296S, is associated with the *Rdl^R^* allele and present only in the two countries with high *Rdl^R^* frequency (Cameroon and Burkina Faso). The V327I mutation has not yet been reported in another mosquito species and it seems conserved between *An. gambiae*, *Cx. quinquefasciatus*, *Ae. aegypti* and *D. melanogaster* (Fig. 2). A similar amino acid replacement in the M2 transmembrane domain from asparagine to lysine was also observed in *Drosophila* at codon 319 (313 in *Anopheles* species) and was responsible for a dramatic increase in conductance (Wang et al., 1999). Further investigations will tell whether or not the V327I mutation in *An. funestus* plays a similar role as the N319K mutation in *Drosophila*.

## 5 Conclusion

The detection of the *Rdl^R^* mutation in *An. funestus* in this study highlights the need to monitor this species for other target-site resistance mutations such as knockdown resistance (kdr) and Ace-1 mutations respectively associated with pyrethroids/DDT and carbamate/organophosphate resistance. As more resistance is reported for this species it is likely that such mutations will be selected in field populations besides the already described metabolic resistance mechanisms.

## Figures and Tables

**Table 1:** Details of pyrosequencing primers and sequence to analyse for *Rdl* mutation.

Primers	Oligo sequences 5′–3′	PCR product
Rdlpyro-F	TCGTGGGTATCATTTTGGCTA	
RdlpyroR-bio	ATGACGAAGCATGTGCCTAA−5′Biotin	167 bp
Rdl-seq	CTACACCAGCACGTGT	
Rdl327seq	TATGTAAAATCGATTG	
Sequence to analyse for A296S	T G/T G/C ATTAGGTGT	
Sequence to analyse for V327I	AC A/G TATATTTAGGC	

**Table 2:** Frequency and genetic parameters of *Rdl^R^* allele in 6 African populations of *An. funestus*.

	*N*	*Rdl^R^* A296S	RR	RS	SS	Fis	*P*	V327I
Burkina Faso	25	0.6	13	4	8	+0.6480	0.0026	0.21
Benin	25	0.16	0	8	17	−0.1707	1	0.0
Cameroon	25	0.82	19	3	3	+0.6066	0.0115	0.175
Uganda	20	0.075	0	3	17	−0.0556	1	0.0
Malawi	25	0.00	0	0	25	/	1	0.0
Mozambique	25	0.00	0	0	25	/	/	0.0

Fis indicates excess (Fis<0) or deficit (Fis>0) of heterozygotes in each sample. P is the probability of a deviation from expectations (bold when *P* < 0.05).
